# The clinical significance of intracranial pressure waveform analysis in brain-injured patients

**DOI:** 10.62675/2965-2774.20260081

**Published:** 2026-03-26

**Authors:** Sérgio Brasil, Fabio Silvio Taccone

**Affiliations:** 1 Universidade de São Paulo Faculdade de Medicina Department of Neurology São Paulo SP Brazil Department of Neurology, Faculdade de Medicina, Universidade de São Paulo - São Paulo (SP), Brazil.; 2 Université Libre de Bruxelles Hôpital Universitaire de Bruxelles Department of Intensive Care Brussels Belgium Department of Intensive Care, Hôpital Universitaire de Bruxelles, Université Libre de Bruxelles - Brussels, Belgium.

**Keywords:** Neurophysiological monitoring, Intracranial pressure, Intracranial hypertension, Brain injuries, Homeostasis

## Abstract

Intracranial pressure monitoring is a cornerstone in neurocritical care, particularly for patients with acute brain injury. Historically, management has focused on absolute intracranial pressure thresholds, but a paradigm shift is underway towards dynamic assessment of intracranial compliance through intracranial pressure waveform analysis. This short review synthesizes recent advancements in understanding intracranial compliance pathophysiology, explores sophisticated invasive and non-invasive monitoring techniques, and discusses their evolving clinical implications. We highlighted how parameters derived from intracranial pressure waveforms, such as the P2/P1 ratio, pulse shape index, and mean pulse amplitude, provide granular insights into the brain compensatory reserve and cerebrovascular autoregulation. Integrating these new pathophysiological insights with advanced monitoring tools holds immense potential to refine clinical decision-making, enabling more proactive, personalized interventions to improve outcomes for patients with acute brain injury.

## INTRODUCTION

Acute brain injuries (ABI), including traumatic brain injury (TBI) and subarachnoid hemorrhage (SAH), pose significant global health challenges. Secondary brain injuries, often driven by intracranial hypertension, profoundly impact morbidity and mortality in these patients. Impairment of intracranial compliance (ICC), defined as the brain's ability to accommodate volume changes without significant intracranial pressure (ICP) elevation, may also contribute to worsening patient outcomes.^(1,2)^ While traditional management of ABI patients relies on absolute ICP values and predefined intervention thresholds, this approach often provides an incomplete picture of underlying intracranial dynamics.^(3)^ There is a growing consensus that a more nuanced, continuous, and dynamic assessment of ICP together with ICC is essential for a better understanding of ABI pathophysiology and individualized patient management.^(4)^ This article aimed to provide an updated summary on the clinical utility of ICP waveform analysis to assess ICC.

## THE EVOLVING UNDERSTANDING OF INTRACRANIAL DYNAMICS

The foundational understanding of ICC stems from the Monro-Kellie doctrine, which posits that the cranial vault is a rigid box containing three main components: brain parenchyma, cerebrospinal fluid (CSF), and arterial/venous blood.^(3)^ Volume changes in one component necessitate compensatory changes (i.e., caudal redistribution of CSF to the spinal subarachnoid space and a reduction in intracranial venous blood volume) to prevent ICP rise. Modern neuroscience recognizes that intracranial dynamics are far more complex. This classical doctrine has integrated the dynamic roles of CSF circulation, including the impairment of the glymphatic system, which can lead to CSF stagnation and altered brain water content, directly impacting ICC.^(3)^ Additionally, parenchymal shrinkage has been identified as an active compliance mechanism, reflecting tissue-level changes that help buffer ICP rises beyond standard pathways.^(5)^ Progressive failure of these buffering mechanisms results in a marked reduction in ICC, which has been defined as the "intracranial compartment syndrome"; at this stage, even minimal additional volume increments produce disproportionate increases in ICP, reflecting a reduced tolerance to minimal changes of intracranial homeostasis.^(6)^

## INVASIVE INTRACRANIAL PRESSURE WAVEFORM ANALYSIS: A WINDOW INTO COMPENSATORY RESERVE

**Figure 1:**
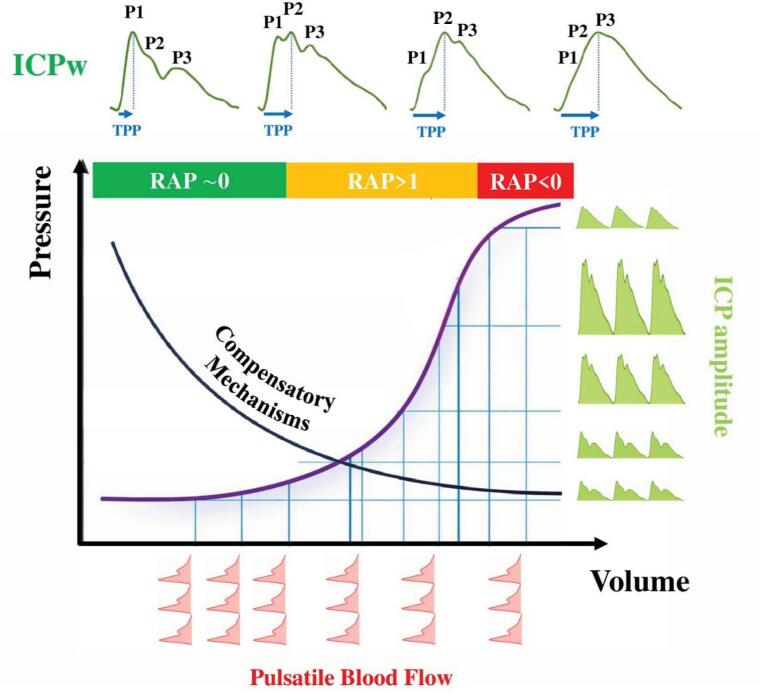
The upper panel illustrates representative intracranial pressure waveforms at different stages of intracranial compliance. Under physiological conditions, the percussion wave (P1), reflecting arterial pulsation transmitted from the choroid plexus, exceeds the tidal wave (P2), which is influenced by intracranial compliance, and the dicrotic wave (P3), associated with aortic valve closure. As compliance deteriorates, P2 progressively increases relative to P1 (P2 > P1), indicating reduced buffering capacity of the intracranial compartment. The middle panel depicts the nonlinear pressure-volume relationship of the brain, highlighting the transition from preserved compensatory reserve (flat segment) to reduced compliance (steep exponential rise in intracranial pressure for minimal volume increments). The amplitude-pressure index (correlation between mean intracranial pressure and pulse amplitude) across stages of compensation: amplitude-pressure index ≈ 0 with intact reserve, amplitude-pressure index approaching +1 with exhausted reserve, and declining amplitude-pressure index values when cerebrovascular reactivity becomes impaired.

A typical ICP waveform comprises three characteristic peaks: P1 (percussion wave), P2 (tidal wave), and P3 (dicrotic wave)*.* The morphology of these pulse waves provides critical information about ICC. As ICC deteriorates, the P2 peak tends to increase relative to P1, leading to a P2/P1 ratio greater than 1.^(7)^ Similarly, the ICP waveform morphology evolves from an almost right-angled triangular pattern to a pyramidal configuration. The time-to-peak also reflects changes in intracranial volume and compliance^(8)^ (Figure 1). The amplitude of the ICP pulse waveform, often referred to as mean pulse amplitude or ICP pulsatility, can also be a useful surrogate of ICC. Indeed, under physiological conditions and when ICC is preserved, the pulsatile arterial inflow during each cardiac cycle induces small buffered ICP oscillations; as compensatory reserve becomes exhausted, identical arterial volume changes generate progressively larger ICP pulsations.^(9)^

ICPw - intracranial pressure waveform; TPP - time-to-peak. RAP - amplitude-pressure index; ICP - intracranial pressure.

Advanced algorithms can further improve ICP waveform analysis. The pulse shape index (PSI), a parameter based on machine learning evaluation, classifies entire ICP pulse wave morphologies into four categories, representing incremental states of compliance.^(2,10)^ Pulse shape index variations can precede subsequent ICP surge, suggesting its potential as an early warning sign.^(1)^ The amplitude-pressure index (RAP) represents the non-linear relationship between mean ICP and pulse amplitude and quantifies the dynamic changes of ICC; a RAP approaching +1 signifies impaired ICC, reflecting the diminishing buffering ability of the brain.^(11)^

## NONINVASIVE APPROACHES FOR INTRACRANIAL PRESSURE WAVEFORM ANALYSIS

The limitations of invasive monitoring (e.g., infection risk, cost) have spurred the development of non-invasive methods for ICC assessment. In particular, the Brain4Care (B4C) system is a non-invasive technology that uses an external wearable mechanical sensor placed on the patient's scalp to detect nanometric skull deformations that occur with each cardiac cycle as intracranial blood volume and pressure fluctuate.^(1)^ These very small skull expansions are captured as a surrogate ICP waveform in real time and generate a P2/P1 ratio and normalized time-to-peak values.^(1)^ Validation studies have demonstrated a strong correlation between B4C-derived parameters (i.e., P2/P1 ratio and time-to-peak) and their homologous invasively measured counterparts.^(12,13)^ Using mean pulse amplitude derived from B4C, the noninvasive pulse amplitude index, a moving correlation coefficient between spontaneous variations in mean arterial pressure and concurrent changes in the non-invasive mean pulse amplitude of the ICP waveform, has demonstrated strong correlation with the invasively calculated pulse amplitude index, supporting its potential role in the non-invasive evaluation of cerebrovascular autoregulation.^(14)^ Moreover, the noninvasive pulse amplitude index has demonstrated meaningful agreement with established autoregulation indices derived from invasive ICP monitoring, supporting its utility as a dynamic surrogate marker of cerebrovascular autoregulatory status.^(13,15)^

## CLINICAL IMPLICATIONS AND FUTURE DIRECTIONS

Advanced interpretation of the ICP waveform should expand bedside neuromonitoring beyond reliance on absolute ICP values alone. Automated extraction of the P2/P1 ratio from invasive ICP signals enables continuous appraisal of ICC and may detect deterioration of compensatory reserve before the development of overt intracranial hypertension. Reliable assessment of mean pulse amplitude, high-fidelity waveform acquisition at the trough, and integration with automated PSI analysis may facilitate early recognition of progressive alterations in intracranial dynamics.

Prospective studies are warranted to determine whether combining waveform-derived metrics with mean ICP improves individualized management. Patients with normal mean ICP but abnormal P2/P1 ratio, increased mean pulse amplitude, or altered PSI may have impaired ICC and could benefit from early corrective measures (e.g., optimizing positioning or avoiding sedation interruptions) or targeted interventions when structural pathology is present. Also, impaired ICC could be used to predict underdrainage in patients with external or implanted shunts for CSF disorders.

Technical considerations are essential: signal artifacts, blood pressure, and carbon dioxide may affect ICP waveform interpretation, PSI is influenced by age, and non-invasive technologies, such as B4C, are not universally available. Moreover, ICP waveform-derived indices may behave differently in patients undergoing decompressive craniectomy.

## CONCLUSIONS

Intracranial pressure waveform analysis provides a dynamic assessment of intracranial compliance and compensatory reserve in brain-injured patients, moving beyond reliance on static intracranial pressure thresholds. By integrating automated indices, such as P2/P1, time-to-peak, or mean pulse amplitude, clinicians can adopt a more individualized and physiology-guided approach to brain-injured patients.

## Data Availability

The contents underlying the research text are included in the manuscript.
