# Erratum: The clinical significance of intracranial pressure waveform analysis in brain-injured patients

**DOI:** 10.62675/2965-2774.20260081err

**Published:** 2026-05-05

In the article **The clinical significance of intracranial pressure waveform analysis in brain-injured patients**, with DOI number: https://doi.org/10.62675/2965-2774.20260081 published in the journal Critical Care Science, 2026;38:e20260081, on page 4, item Conflicts of interest:

**Where it read:**

None.

**Read:**

Dr. Sergio Brasil is a senior scientific advisor for Brain4Care.

**Responsible editor:** Jorge Ibrain Figueira Salluh

